# The Differential Diagnosis of Abdominal Mass: A Case of Uterine Leiomyoma

**DOI:** 10.7759/cureus.65126

**Published:** 2024-07-22

**Authors:** Shivangi Ghildiyal, Savita Somalwar, Anuja Bhalerao, Sheela Jain

**Affiliations:** 1 Department of Obstetrics and Gynecology, Narendra Kumar Prasadrao (NKP) Salve Institute of Medical Sciences and Research Centre, Nagpur, IND

**Keywords:** intramural leiomyoma, abnormal uterine bleeding, exploratory laparotomy, hysterectomy, uterine fibroids, benign leiomyoma

## Abstract

The most common benign neoplastic uterine tumors that grow monoclonally from the smooth muscle cells of the uterus are uterine fibroids or leiomyomas, which may occur as a single lesion or as multiple lesions with variation in size from microscopic to large macroscopic extent. The majority are diagnosed in the preclinical routine reliably, despite challenges due to the possibility of multiple differential diagnoses. Hence, this report highlights a case of a postmenopausal female of 53-year-old working as a staff nurse at the same hospital and who visited the outpatient department of obstetrics and gynecology with chief complaints of pain in the right side of the abdomen for four hours (presentation similar to that of appendicitis). Per abdomen examination resulted in a non-tender mass with flank fullness and firmness with a smooth surface and approachable lower border. It clinically appeared as a large uterine fibroid. The built of the patient was obese due to which neither the patient nor relatives were ever able to make out any evident symptoms. The diagnostic investigation involved a magnetic resonance imaging (MRI) that confirmed the diagnosis of two parity with both living, and two were aborted (P2L2A2) with uterine fibroid. The management of the fibroid consisted of exploratory laparotomy along with a hysterectomy and bilateral salpingo-oophorectomy. The intraoperative findings and frozen section report confirmed the presence of benign uterine leiomyoma. Therefore, the utilization of physical examinations and diagnostic tests may assist in preventing a delay in the detection and management of curable conditions such as fibroids, which can be treated without complications with surgery.

## Introduction

The most prevalent benign neoplastic uterine tumors are leiomyomas or uterine fibroids. Due to hormone-stimulated growth, they affect 20%-30% of women in the reproductive phase between the ages of 30 and 50 that grow from the smooth muscle cells of the uterus monoclonally. However, they are found to be less common in adolescents with less than 1% incidence [1,2]. Most patients require only conservative care because they are either asymptomatic or just have minimal symptoms [3]. Many risk factors, including genetic changes, positive family history, and lifestyle choices, have been linked to the formation of these tumors. At the same time, the underlying pathophysiology of their development is still unknown [4,5]. In two-thirds of cases, myomas have been observed to appear as numerous lesions ranging in size from microscopic to huge macroscopic. The various types of fibroids involve subserosal, which consists of fibroid projecting to the outside of the uterus; intramural, in which the fibroid grows within the muscular uterine wall; and submucosal, in which the fibroid bulges into the uterine cavity [6]. Undiagnosed uterine fibroids are common since most women with myomas do not exhibit any symptoms. Women with symptoms most commonly experience dysmenorrhea and abnormal uterine bleeding (polymenorrhea and metro- or menorrhagia). Acyclic chronic pelvic pain and dyspareunia are considered the other frequent symptoms [4-8].

As fibroids (mostly submucosal and intramural) interfere with fertility [9], they can severely affect women's psychological health [5]. The several mechanisms involved consist of abnormal vascularization, chronic inflammation, increased uterine contractility, and deranged cytokine profile [10,11]. Myomas can result in symptoms related to compression, such as frequent urination, dyspnea, or gastrointestinal issues, due to continued growth. Myomas grow at different rates within and across individuals, which causes them to either regress or gradually enlarge until the climacteric period is possible. Ultrasound tests with attentive observation are necessary to diagnose rapidly growing and progressing fibroids. Additionally, degenerative changes observed consist of myxomatous changes, fatty changes, mucoid changes, hyaline degeneration, cystic degeneration, red degeneration, and calcification. These changes can lead to malignant transformation in postmenopausal older and middle-aged females and therefore should be considered during progressive enlargement of the fibroid [11]. Large myomas can have serious side effects, such as diaphragmatic compression leading to the failure of the respiratory system and an incarcerated hernia in the abdominal wall [2,12]. The majority of uterine myomas are accurately identified in the preclinical settings, although diagnosing a huge myoma is challenging because of the many potential differential diagnoses. Hence, this report highlights a case of benign leiomyoma in a 53-year-old female.

## Case presentation

Patient information

A 53-year-old female reported to the outpatient department of obstetrics and gynecology with chief complaints of pain in the right side of the abdomen for four hours. The obstetric history described by the patient involved two parity with both living, and two were aborted (P2L2A2). Both abortions were induced and were performed at three months and two months of the respective years. Additionally, the menstrual history stated that the patient was postmenopausal with abnormal uterine bleeding for two years. Moreover, the patient reported no significant family or personal history.

Clinical examination

The patient was vitally stable, and the systemic examination was also found to be normal. Per abdomen examination resulted in a mass corresponding to a 36-week gravid uterus with flank fullness and firmness. The mass was non-tender, with a smooth surface and an approachable lower border. Per speculum examination reported the cervix flushed with the vagina and was short (0.5 cm) and multiparous, and per vaginal examination illustrated that the cervix was pinpoint and was taken up. Laboratory investigations revealed no significant findings, and CA125 was 43.23 u/mL. Additionally, a pap smear examination revealed an inflammatory smear with reactive cellular changes.

Diagnostic assessment

**Figure 1:**
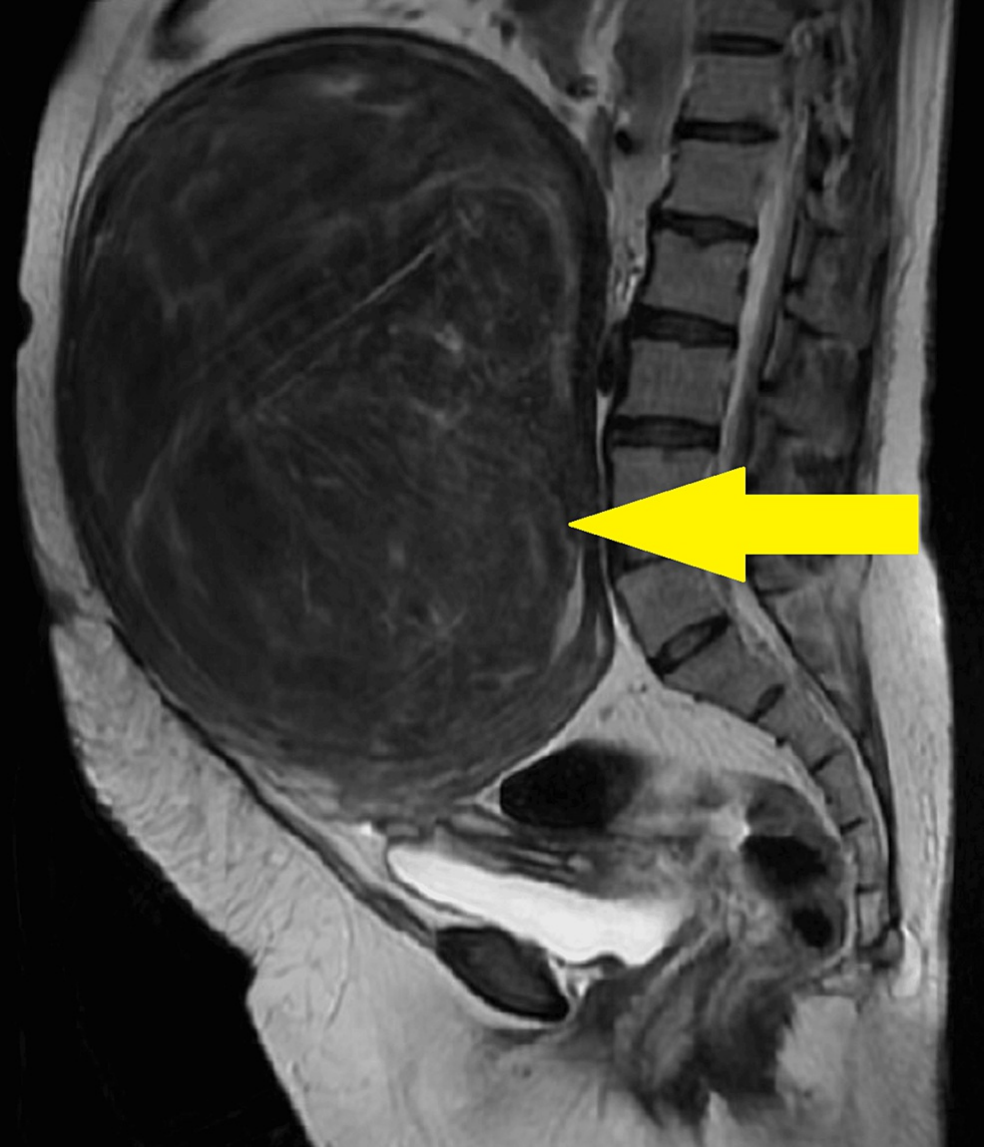
Magnetic resonance imaging illustrating uterine fibroid Yellow arrow: a large, well-defined heterogeneous altered signal intensity lesion in the anterior myometrium of the uterus that measured 16.5 × 26.6 × 24.1 cm and pushed the endometrial thickness posteriorly extending from the upper border of T12 to S1 vertebra

For further investigations, diagnostic assessment consisting of magnetic resonance imaging (MRI) reported a large, well-defined heterogeneous altered signal intensity lesion in the anterior myometrium of the uterus that measured 16.5 × 26.6 × 24.1 cm and pushed the endometrial thickness posteriorly extending from the upper border of T12 to S1 vertebra as illustrated in Figure 1. The endometrial thickness measured was 4.5 mm. Based on the abovementioned findings, the diagnosis of P2L2A2 with uterine fibroid was confirmed, and the patient was advised for exploratory laparotomy with hysterectomy.

Therapeutic intervention

**Figure 2:**
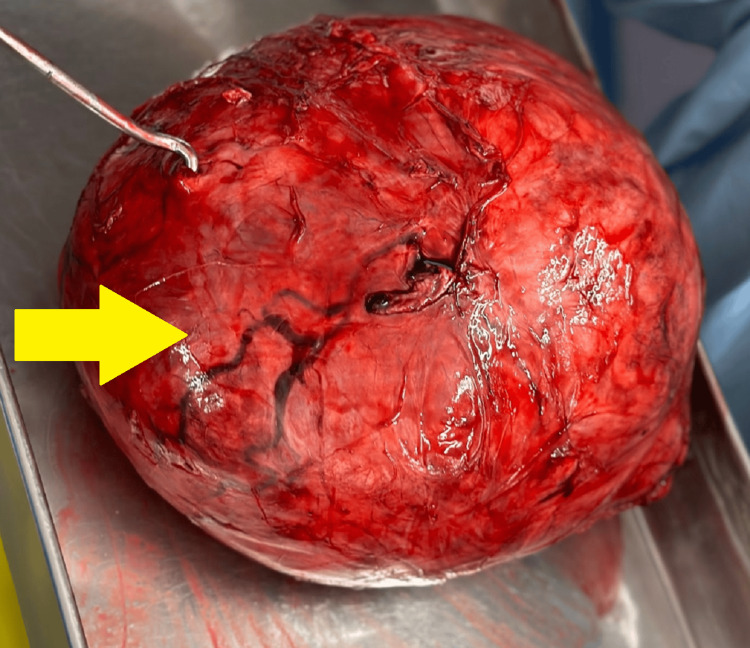
Benign cellular leiomyoma Yellow arrow: a large, highly vascular uterine fibroid that was fundal and intramural of size 24 × 20 × 18 cm with a weight of 5.78 kg

The patient was advised to execute exploratory staging laparotomy with hysterectomy for which required written informed consent was obtained before commencement of the procedure. The procedure for staging laparotomy was performed under all aseptic precautions under general anesthesia with the patient in a supine lying position. Betadine painting and draping were done, and a midline vertical incision was made extending from the pubic symphysis to 7-8 cm above the umbilicus. The abdomen was opened layer by layer up to the peritoneum, and peritoneal fluid was collected and sent for cytology. A large, highly vascular uterine fibroid that was fundal and intramural of size 24 × 20 × 18 cm with a weight of 5.78 kg was observed as illustrated in Figure 2.

Additionally, the uterus was enlarged to 36 weeks size with a weight of 1.3 kg. The fibroid was exteriorized, and omental adhesions were noted, clamped, and cut. Bilateral round and tubo-ovarian ligaments were clamped, cut, and transfixed. The bladder was separated using blunt and sharp dissection and was pushed down. The left-sided uterine artery was clamped, cut, and ligated. Myoma was instilled with the normal saline, followed by the insertion of the myoma screw, and traction was given. A myomectomy was performed, and the sample was sent for a frozen section. The right-sided uterine artery was clamped, cut, and ligated. Bilateral uterosacrals and Mackenrodts were clamped, cut, and transfixed, and the vault was opened. The uterus with bilateral fallopian tubes was delivered out completely. The vault was deep-seated, and the subtotal hysterectomy was performed as the fibroid was symptomatic and the patient did not want to have further children. Moreover, the cervix along with the ligaments that support it maintains normal anatomical support of the pelvis and therefore was not removed consequently, leading to less chance of urinary incontinence and vaginal vault prolapse. The specimen was taken out, and the vault was closed. Additionally, cysts were observed in bilateral ovaries, and therefore, the decision of oophorectomy was taken and performed. The bilateral infundibulopelvic ligaments were clamped, cut, and transfixed, and bilateral ovaries were removed completely. Hemostasis was achieved. The abdomen was closed in layers using loop prolene, and the skin was closed using vertical mattress sutures. The frozen section report confirmed the diagnosis of benign cellular leiomyoma. The patient received antibiotics and analgesics. The postoperative period was uneventful. Sutures were removed on day 12, and the patient was discharged. The final histopathology report was suggestive of cellular leiomyoma.

## Discussion

The present case involved a 53-year-old female with a benign uterine leiomyoma or fibroid that was fundal and intramural of size 24 × 20 × 18 cm with a weight of 5.78 kg. For diagnostic purposes, an MRI was performed, and based on the findings, the diagnosis was confirmed. The management of the fibroid consisted of exploratory laparotomy along with a hysterectomy and medicinal intervention. Moreover, the intraoperative findings and frozen section report confirmed the presence of benign leiomyoma. The diagnosis is further complicated by dystrophic calcification and the possibility of the benign tumors changing.

Misdiagnoses of uterine leiomyomas include pregnancy, hematometra, adenomyosis, ovarian tumors, and uterine sarcoma [2,13]. Other typical differential diagnoses that are not related to gynecology are inflammation or gastrointestinal tumors [14]. Fibroids frequently coexist with adenomyosis and endometriosis, exhibiting overlapping symptoms [15], which significantly lowers the confidence in the diagnosis. The patient's symptoms and the accuracy of the diagnosis are influenced by the location of the fibroid in the uterus. Another differential diagnosis is uterine cancer, of which carcinomas are the most prevalent type. Sarcomas and carcinosarcomas, however, are uncommon [8]. Of all cases, 0.2% involve the malignant conversion of a leiomyoma to a leiomyosarcoma [16].

Significantly larger fibroids cannot be treated with the most common minimally invasive surgical techniques, which corresponds with the present case as there is a chance of conversion to laparotomy, complication rates, and the surgeon's limited experience. Depending on the patient's age and both the adnexa affection, the majority of the giant fibroids were removed during a total abdominal hysterectomy with additional bilateral salpingo-oophorectomy [8,17]. Patients who undergo a laparotomy have a significantly greater overall morbidity and mortality rate relative to the amount of surgery. The clinical gold standard for women with progressing myomas is early surgical therapy along with attentive surveillance to prevent the development of giant fibroids. In Germany, in 60.7% of all cases, surgical hysterectomy is indicated for uterine fibroids [2,15].

The frozen section has a 96% overall specificity for the diagnosis of leiomyomas, with negligible false-positive and false-negative rates. Ensuring the target tissue sample and collecting a sufficient volume of tissue is one of the frozen section's limitations [1,18]; therefore, to provide an adequate sample for the most accurate diagnosis, in the present case, the entire specimen was sent for histopathological examination. However, surgical challenges were expected that involved intraoperative determination of anatomy, access, and hemorrhage. Moreover, both young and experienced surgeons encounter challenges with such large masses involving uncertain diagnoses.

## Conclusions

The present case report demonstrated a case of an elderly female with a large mass arising from the fundus and the body of the uterus, which on diagnosis confirmed the presence of benign uterine leiomyoma. Therefore, utilization of physical examination and diagnostic tests at appropriate duration may assist in preventing a delay in the detection and management of curable conditions such as fibroids. Additionally, preclinical utilization of the services of gynecologists depends on various factors that involve the educational level, family background, and socioeconomic status of the patient, which needs to be taken into consideration. Moreover, giant fibroids continue to be a surgical and diagnostic challenge requiring multidisciplinary collaboration and specialized expertise. Nevertheless, with the right diagnosis and skilled surgery, even enormous benign tumors can be treated without experiencing any complications.
